# Tuning the MYC response

**DOI:** 10.7554/eLife.18871

**Published:** 2016-07-27

**Authors:** Ying Zheng, David Levens

**Affiliations:** Laboratory of Pathology, Center for Cancer Research, National Cancer Institute, Bethesda, United States; Laboratory of Pathology, Center for Cancer Research, National Cancer Institute, Bethesda, United Stateslevensd@mail.nih.gov

**Keywords:** MYC, promoter affinity, ChIP-sequencing, mathematical modeling, WDR5, MIZ1, Human, Mouse

## Abstract

Altering the ability of the MYC transcription factor to bind to individual genes can customize the global gene expression output of cells.

**Related research article** Lorenzin F, Benary U, Baluapuri A, Walz S, Jung LA, von Eyss B, Kisker C, Wolf J, Eilers M, Wolf E. 2016. Different promoter affinities account for specificity in MYC-dependent gene regulation. *eLife* **5**:e15161. doi: 10.7554/eLife.15161

**Image** High levels of MYC protein in cells can promote the growth of tumors

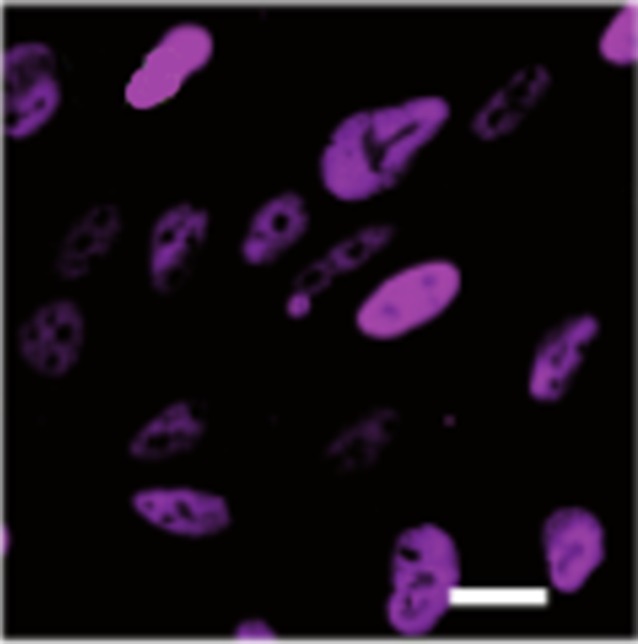

Cancer develops when cells in the body gain mutations that enable them to divide rapidly and form a tumor. Some of these mutations can increase the expression of particular genes in cells. For example, a gene that encodes a transcription factor protein called MYC is upregulated in most types of tumor, where it supports several aspects of tumor development.

Transcription factors can bind to promoter regions at the start of genes to regulate the first step in gene expression, a process called transcription. MYC can bind to virtually all promoters in the genome and trigger widespread increases in gene activity. However, it is not clear whether MYC binding directly increases the transcription of all the genes (Levens, 2013; Lin et al., 2012; Nie et al., 2012; Wolf et al., 2015), or whether MYC only regulates specific sets of genes that then increase the expression of other genes (Kress et al., 2015).

Previous studies have shown that producing more MYC in cell lines does not increase the expression of all genes equally (Lin et al., 2012; Sabò et al., 2014; Walz et al., 2014). Now, in eLife, Elmar Wolf at the University of Würzburg and colleagues – including Francesca Lorenzin as first author – show that changing the amount of MYC in cells does not change the amount of MYC bound at each gene in the same way (Lorenzin et al., 2016). This prompted the researchers to seek an explanation for the relationship between MYC levels and gene expression across all genes.

Lorenzin et al. – who are based at the University of Würzburg and the Max-Delbrück-Center for Molecular Medicine – manipulated the levels of MYC in a human cell line called U20S, which normally has less of this protein compared to many cancer cell lines. Upregulating MYC in U2OS cells to match the levels seen in cancer resulted in MYC molecules being bound to virtually all promoters.

Further experiments indicated that MYC binding to any promoter can alter the activity of the corresponding gene, but that MYC is better at binding to some promoters than others. At normal levels of MYC, promoters where MYC binds weakly (i.e. those with a low binding affinity) will have few MYC molecules bound to them. However, under the same conditions, high-affinity promoters may already be saturated with MYC molecules. If cells make more MYC, the number of MYC molecules binding to the lower-affinity promoters would increase, but the high-affinity promoters would be largely unaffected. Therefore, the increases in MYC production observed in cancer cells can alter the transcription of some genes more than others. Because every cancer starts with a different pattern of gene expression and a different level of MYC, this may result in each tumor having a distinct transcription profile.

What determines how strongly MYC binds to a promoter? Lorenzin et al. modeled MYC binding across the genome, taking into account that MYC is known to bind strongly to a DNA motif called the E-box (Guo et al., 2014), and less tightly to other DNA sequences (Figure 1). But the model based on E-boxes alone did not explain how MYC is distributed across the genome. Lorenzin et al. then considered the possibility that interactions between MYC and other proteins may help to draw MYC to the promoters of active genes. For example, a protein called WDR5 was speculated to help MYC target genes (Thomas et al., 2015). Indeed, including interactions between MYC and WDR5 markedly improved the model for many (but not all) genes. Moreover, impairing this interaction had a greater impact on the expression of genes with high affinity for MYC than those with low affinity.

**Figure 1.**
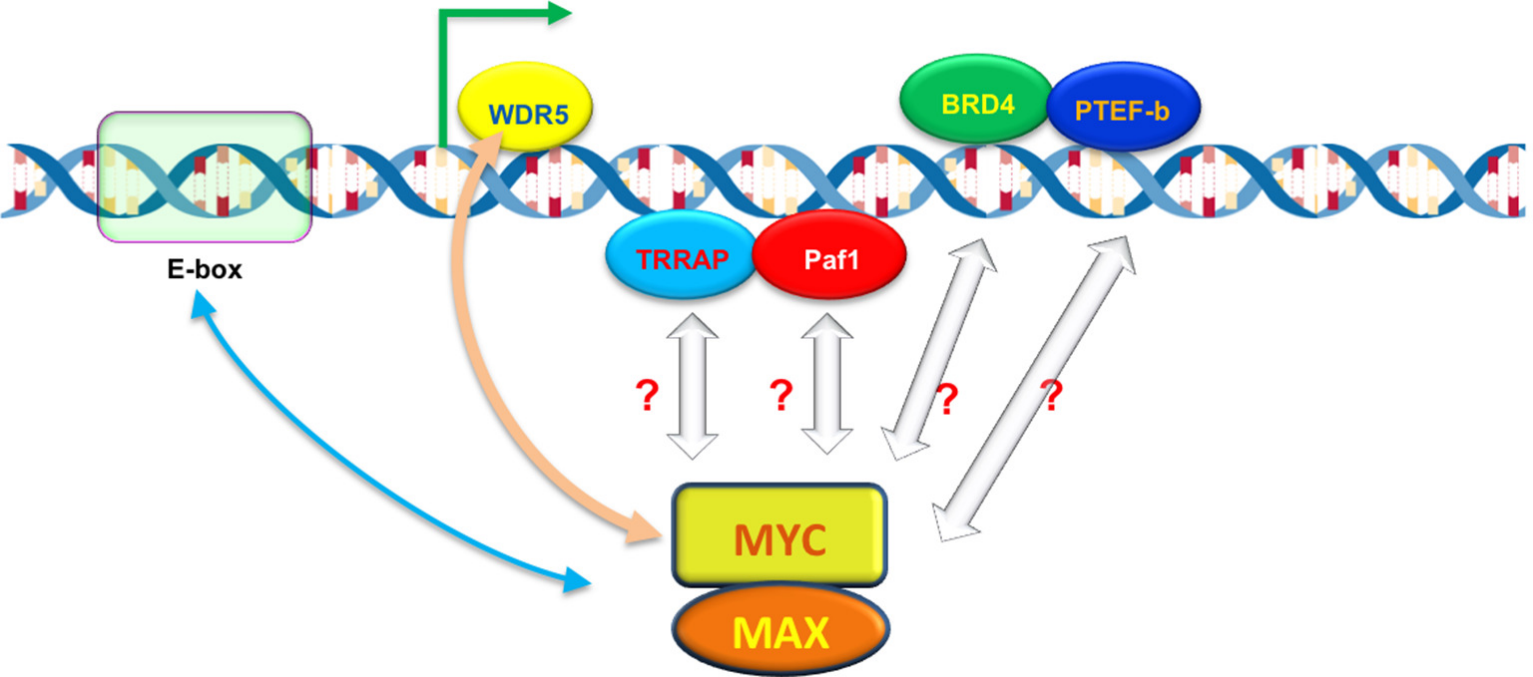
The ability of MYC to bind to individual promoters is influenced by interactions with DNA and other proteins. The MYC protein forms a dimer with another protein called MAX, which allows it to bind to virtually any promoter in the genome and increase gene expression by activating transcription (green arrow). However, the ability of MYC to bind to individual promoters (binding affinity) varies. MYC prefers to bind to DNA motifs called E-boxes, but will also bind to other DNA sequences with lower affinity. A protein called WDR5, which is associated with the DNA of active genes, can help to recruit MYC to particular promoters (beige arrow). MYC also interacts with other DNA-associated proteins – including TRAPP, Paf1, BRD4 and PTEF-b – during transcription, which may also help MYC to bind to certain promoters with higher affinity.

Besides WDR5, MYC interacts with many other proteins and protein complexes during transcription (Figure 1). Do these interactions also help to recruit and retain MYC at promoters, and if so, how might they be incorporated into the model? Perhaps MYC interacts with different partners in different stages of transcription to ensure that the process happens in an efficient and orderly manner. It should be noted that some of these partners might trigger the degradation or modification of MYC leading to its ejection from certain promoters, which would change its apparent binding affinity, and in turn change the outputs of these promoters.

Finally, normal cells contain less MYC than the cells used by Lorenzin et al. and so the majority of promoters in a normal cell are not saturated with MYC molecules. In cancer cells, the genes with the highest affinity for MYC, such as the ribosomal protein genes, are saturated with MYC and are also the most sensitive to its inhibition or reduction. Understanding exactly how MYC binding influences gene expression promises to aid efforts to develop new therapeutic strategies that inhibit its cancer-promoting actions, while preserving its normal responsibilities in cells.
